# The Many Landscapes of Recombination in *Drosophila melanogaster*

**DOI:** 10.1371/journal.pgen.1002905

**Published:** 2012-10-11

**Authors:** Josep M. Comeron, Ramesh Ratnappan, Samuel Bailin

**Affiliations:** 1 Department of Biology, University of Iowa, Iowa City, Iowa, United States of America; 2 Interdisciplinary Program in Genetics, University of Iowa, Iowa City, Iowa, United States of America; Stanford University, United States of America

## Abstract

Recombination is a fundamental biological process with profound evolutionary implications. Theory predicts that recombination increases the effectiveness of selection in natural populations. Yet, direct tests of this prediction have been restricted to qualitative trends due to the lack of detailed characterization of recombination rate variation across genomes and within species. The use of imprecise recombination rates can also skew population genetic analyses designed to assess the presence and mode of selection across genomes. Here we report the first integrated high-resolution description of genomic and population variation in recombination, which also distinguishes between the two outcomes of meiotic recombination: crossing over (CO) and gene conversion (GC). We characterized the products of 5,860 female meioses in *Drosophila melanogaster* by genotyping a total of 139 million informative SNPs and mapped 106,964 recombination events at a resolution down to 2 kilobases. This approach allowed us to generate whole-genome CO and GC maps as well as a detailed description of variation in recombination among individuals of this species. We describe many levels of variation in recombination rates. At a large-scale (100 kb), CO rates exhibit extreme and highly punctuated variation along chromosomes, with hot and coldspots. We also show extensive intra-specific variation in CO landscapes that is associated with hotspots at low frequency in our sample. GC rates are more uniformly distributed across the genome than CO rates and detectable in regions with reduced or absent CO. At a local scale, recombination events are associated with numerous sequence motifs and tend to occur within transcript regions, thus suggesting that chromatin accessibility favors double-strand breaks. All these non-independent layers of variation in recombination across genomes and among individuals need to be taken into account in order to obtain relevant estimates of recombination rates, and should be included in a new generation of population genetic models of the interaction between selection and linkage.

## Author Summary

Most sexual eukaryotes require recombination between homologous chromosomes for the proper formation of haploid gametes from diploid germ cells. Evolutionarily, recombination increases the effectiveness of selection in natural populations, thus explaining the pervasiveness of recombination and sex. Recombination is also a central parameter in population genetic studies designed to detect the presence of selection acting across genomes. Current evolutionary analyses are hindered, however, by the use of imprecise recombination rates that can influence the results and skew their interpretation. This limitation is associated with the lack of detailed characterization of natural variation in recombination across genomes and within species. Our study in *Drosophila melanogaster* represents the first integrated, whole-genome description of recombination that alleviates these deficiencies in any organism. Our results and conclusions will help to characterize the molecular basis of the observed variation in recombination across genomes and have an immediate impact on population genetic analyses of selection, laying the foundation for a new generation of population models that will better capture natural variation in recombination and its consequences.

## Introduction

Recombination is a fundamental biological process. Mechanistically, most sexual eukaryotes require recombination between homologous chromosomes for proper formation of haploid gametes from diploid germ cells [1]. Evolutionarily, recombination is predicted to increase the effectiveness of selection in natural populations, thus explaining the pervasiveness of recombination and sex [2]–[9]. In most species, recombination rates also vary among and along chromosomes. This intra-genomic variation provides a unique opportunity to evaluate the evolutionary consequences of recombination due to exposure to identical demographic and environmental factors; an *all else being equal* premise that is hardly ever warranted when comparing populations or species. Population genetic analyses across genomes have confirmed the profound effects that recombination imposes on the evolutionary process, shaping levels of genetic variation, limiting the accumulation of deleterious mutations and enhancing rates of adaptation in many species including *Drosophila*, *Arabidopsis thaliana, Saccharomyces cerevisiae* and nematodes [10]–[25]. Yet these studies have been hindered, at least in part, by three limitations associated with the lack of detailed characterization of natural variation in recombination across genomes and within species.

First, population genomic analyses can now analyze patterns of selection at the scale of single genes, or even focus on specific gene regions, but most whole-genome genetic maps localize recombination events with much less detail [24], [26]–[31]. Second, most species' maps based on direct measures of recombination are obtained either from two specific, usually highly diverged, strains (e.g., *Caenorhabditis elegans* [30] or *S. cerevisiae* [32]), or compiled from crosses in different laboratory/natural conditions that can also influence recombination rates [13], [26], [28]. These genetic maps are customarily assumed to represent a monomorphic description of recombination for a given species, even when there is ample evidence of intra-specific natural variation. In species like *A. thaliana*, *D. melanogaster*, *C. elegans*, maize, mice or humans, variation has been reported for the number of recombination events at the level of whole chromosomes [28], [30], [33]–[35] or for specific chromosomal intervals [36]–[44], but a high-resolution whole-genome study of variation in genetic maps based on multiple natural strains is lacking.

Third, the process of meiotic recombination associated with the repair of double strand breaks (DSB) has two possible outcomes with diverse evolutionary consequences: crossing over (CO) and non-crossing over (or gene conversion; GC). Although CO events also include a gene conversion tract, we will use GC to refer to recombination events that are not accompanied by crossing over (Figure S1). Unlike CO, GC results in the exchange of only small tracts of a chromosome, interrupting linkage disequilibrium in a much localized manner while having no effect on linkage disequilibrium at longer intervals, thus GC plays a stronger role than CO at short physical distances [45]–[48]. Corresponding high-resolution CO and GC maps based on direct experimental detection of recombination events however are only available for the unicellular yeast *Saccharomyces cerevisiae* [32]. In multicellular organisms, CO maps are used as proxy for total recombination, and the consequences of GC are overlooked, despite its potential influence on total recombination, particularly in regions or chromosomes with limited CO.

To alleviate all these deficiencies, we generated high-resolution, whole-genome CO and GC maps from eight crosses between natural strains of *D. melanogaster* (see Materials and Methods for details). In order to obtain haploid genomes resulting from female meioses, we crossed heterozygous *D. melanogaster* females to males of *D. simulans* and genotyped the female hybrid progeny using whole-genome high-throughput Illumina sequencing. Reads corresponding to *D. simulans* were removed bioinformatically by mapping them to *D.simulans* genomic sequences. We then generated a whole-genome *D. melanogaster* haplotype per genotyped fly by mapping high-quality informative SNPs to one of the two parental *D. melanogaster* strains. Recombination events were detected, and directly assigned as CO or GC events, based on changes in parental identity along each *D. melanogaster* haplotype.

Overall, we characterized the products of 5,860 female meioses and genotyped an average of 49,000 informative SNPs per fly, for a total of 139 million SNPs. We mapped more than 106,000 recombination events (CO and GC combined) with a median distance to the nearest informative SNP of less than 2.0 kb (1.83 kb). This resolution is almost equivalent to the high-resolution mapping of meiotic recombination in the unicellular *S. cerevisiae* [32], 15-fold higher than the linkage map in *A. thaliana* also based on recombinant inbred lines [27], and more than 50-fold more detailed than current high-resolution whole-genome CO maps in humans [28], *C. elegans* [30], *C. briggsae* [24], or *D. pseudoobscura* [29].

## Results

### A high-resolution CO map for *D. melanogaster*

**Figure 1:**
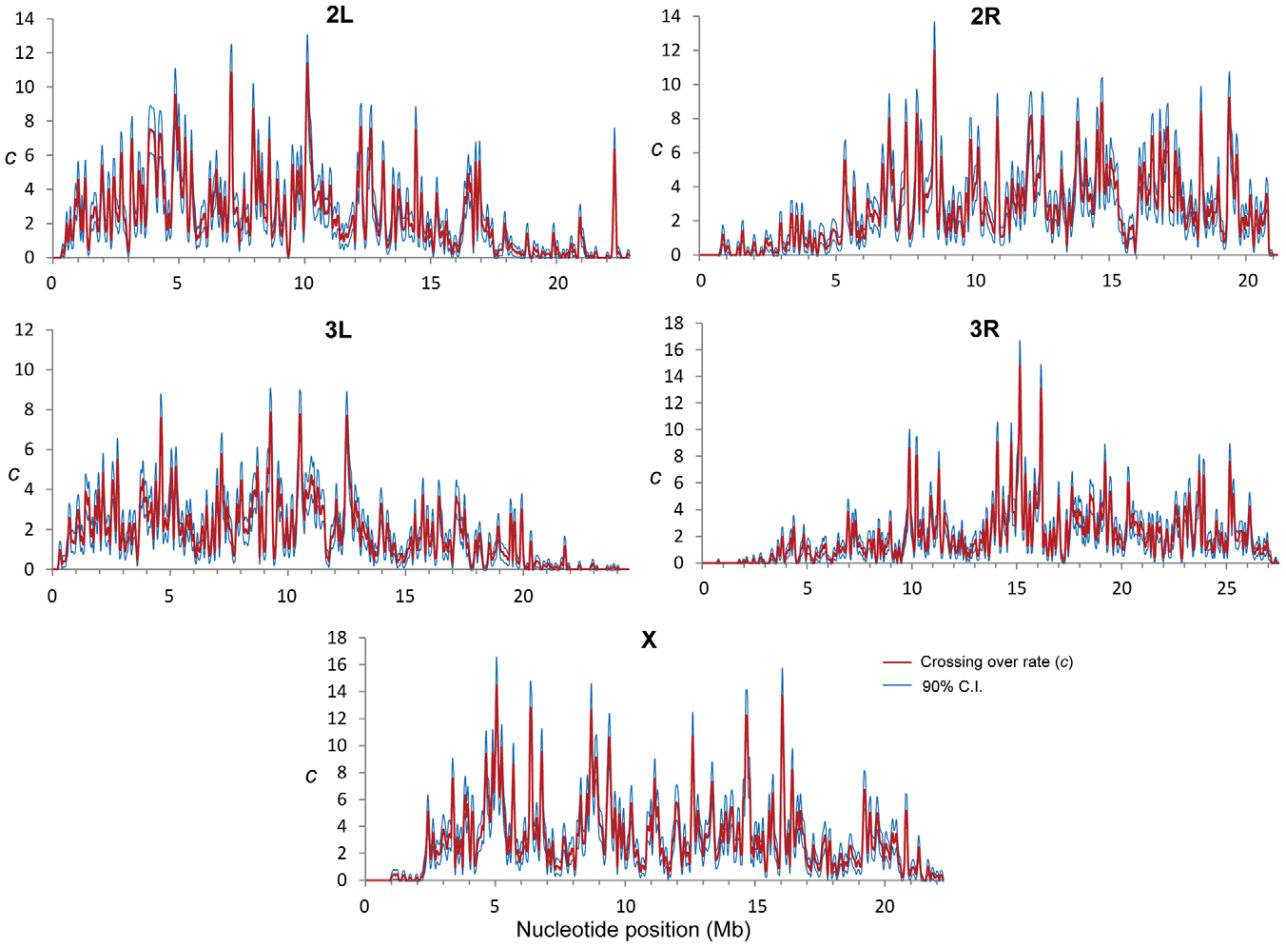
Crossing over rate variation along chromosome arms in *D. melanogaster*. Rate of crossing over (*c*) based on data from all crosses and indicated in centimorgans (cM) per megabase (Mb) per female meiosis (red line). *c* is shown along chromosomes for 100-kb windows and a movement between adjacent windows of 50 kb. Blue lines indicate 90% confidence interval for *c* at each window.

Combining the results from all crosses we detected a total of 32,511 CO events that were used to generate high-resolution CO maps in *D. melanogaster* (Figure 1). Due to the elevated density of markers and the small number of CO events per chromosome and genotyped fly, each CO is supported by many contiguous markers at either side and it is our expectation that we have detected all COs. The total genetic map length for *D. melanogaster* obtained in our crosses is 287.3 cM, closely matching classical measures (282 cM [26]). A low-resolution approximation to the distribution of CO rates (*c*) along chromosome arms based on our data (Figure S2) recovers the same general, large-scale distribution as previous maps based on visible markers [11]–[13], [26], [49]–[53]. As expected, *c* is sharply reduced near telomeres and centromeres, and we detect no CO events in the small fourth (dot) chromosome that proceeds to meiotic segregation without chiasmata [54].

Our detailed maps deepen the recent appreciation for intra-chromosomal variation in CO rates in *Drosophila* [29], [55], [56] and outline this heterogeneity at a much finer scale across the whole genome. Heterogeneity in CO rates along each chromosome is significant at all physical scales analyzed, from 100 kb to 10 Mb, even after removing centromeric and telomeric regions with visibly reduced rates (*P*<0.0001 in all cases; see Materials and Methods). All chromosome arms (except the fourth chromosome) show 15-to-20-fold variation within regions traditionally labeled as regions of non-reduced recombination rates based on low-resolution maps. This heterogeneity in CO rates is highly punctuated, with intense short-distance variation and several adjacent 100-kb windows differing by 15-to-20-fold (eg., region 15.9—16.1 Mb in the X chromosome) thus defining *hot*- and *coldspots* for CO in *D. melanogaster*. Most coldspots are 100-kb regions embedded in larger regions with non-reduced recombination, but we also detect several larger regions that show consistently low CO rates (e.g., a region around position15.8 Mb along chromosome arm 2R) in addition to centromeric/telomeric sequences.

### Intraspecific variation in CO landscapes

**Figure 2:**
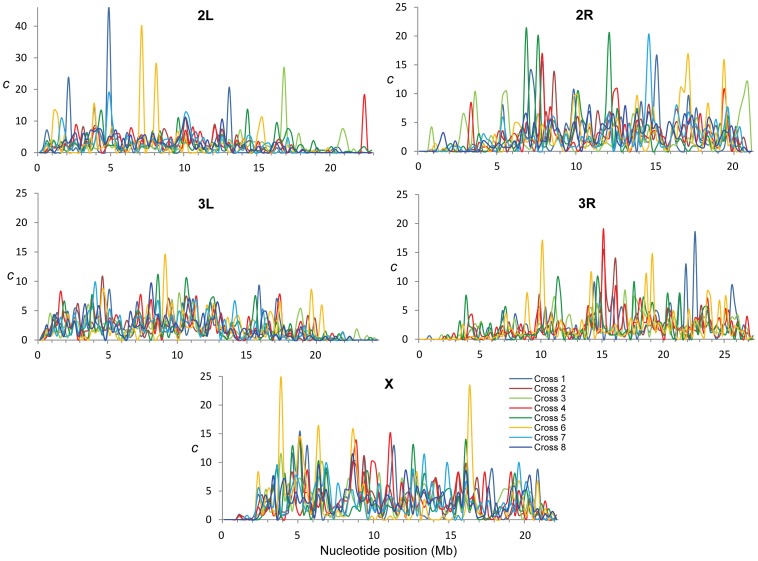
Intra-specific variation in crossing over rates along chromosome arms. *c* (cM/Mb per female meiosis) for eight different crosses (different colors) and shown for adjacent 250-kb windows.

The study of crosses of natural *D. melanogaster* strains allowed us to generate and compare eight CO maps after controlling for variation associated with factors that can alter CO rates in *Drosophila* such as age, temperature, number of matings or food [57]–[60]. To increase statistical power we focused on differences among crosses at the scale of 250-kb along chromosomes. The eight CO maps reveal a high degree of intra-specific variation, with particular crosses having regions with exceedingly high rates (>40-fold) relative to either adjacent regions or to other crosses (Figure 2). As expected, crosses sharing one parental strain have more similar maps than crosses not sharing parental strains but the overall magnitude of the correlation between these crosses, albeit significant, is rather small (Spearman's *R* = +0.451). This observation reinforces the concept of a highly polygenic and polymorphic basis for CO distribution along chromosomes.

**Figure 3:**
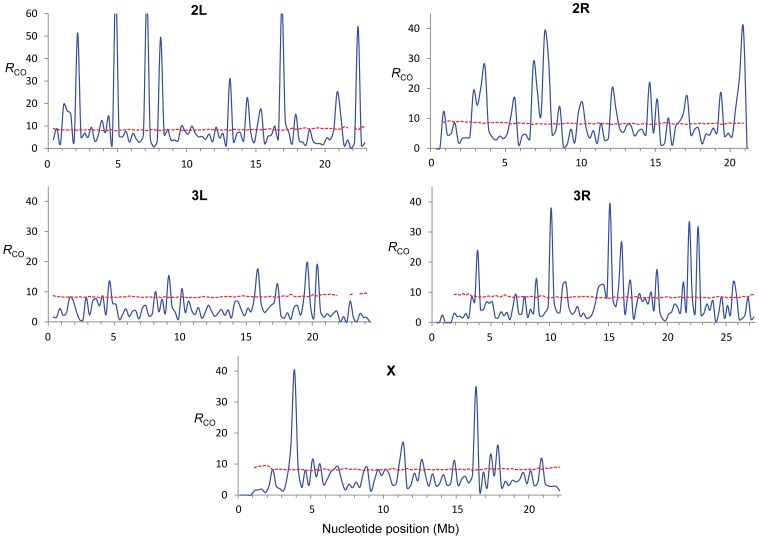
Estimate of the Index of Dispersion (*R*_CO_) for rates of crossing over along chromosome arms. *R*_CO_ was obtained by comparing crossing over rates from eight crosses (see Materials and Methods for details) and is shown for adjacent 250-kb windows (blue line). The doted red line indicates the *P* = 0.0005 confidence threshold (equivalent to *P* ( = 0.05)/number of windows in whole-genome analyses).

To quantify variation in CO rates among the eight CO maps we estimated the variance to mean ratio (Index of Dispersion; *R*_CO_) and tested whether the different number of CO events at a given region can be explained by a Poisson process. Moreover, we focused on variation in the *distribution* of CO rates along chromosomes and therefore we took into account the number of total events for each chromosome (see Materials and Methods for details). Our study of *R*_CO_ along chromosomes reveals many regions (107 or 22% of all non-overlapping 250-kb regions across the genome) with a variance among crosses larger than expected (overdispersion) and this pattern is observed in all chromosomes (Figure 3). The magnitude of this excess variance is highest for chromosome arm 2L while notably reduced for the chromosome arm 3L. Significant overdispersion of CO rates among crosses is also detected when we study larger genomic regions. At a physical scale of 1 Mb, more than half of the genomic regions exhibit excess variance, thus suggesting that regions with variable CO rates are frequent enough across the *D. melanogaster* genome to be playing a detectable role in a large fraction of these longer sequences.

These results are consistent with early studies in *Drosophila* that reported natural variation in CO rates based on artificial selection experiments ([61] and references therein). Our genome-wide study details the genomic location and magnitude of this variation and depicts the first high-resolution polymorphic landscape of CO rates in *D. melanogaster*. Several genomic regions have low rates in all crosses, thus representing monomorphic (or high-frequency) coldspots for CO in *D. melanogaster*. Other regions assigned as peaks of CO rates based on combined maps, however, are strongly influenced by polymorphic hotspots at low frequency in our sample. In fact, most regions with excess variance in CO rates among crosses are associated with low-frequency hotspots rather than low-frequency coldspots suggesting that hotspots are transient (short-lived) features within *D. melanogaster* populations.

Our results thus indicate that CO rates based on multiple crosses and genotypes are needed to obtain a representative depiction of a “species” recombination landscape. Additionally, the low frequency of the hotpots will strongly influence measures of recombination based on the arithmetic mean of all maps, suggesting higher rates than measures such the harmonic mean or median (see Figure S3 for a comparison between mean and median CO values). Notably, we observe genomic regions with very low (or zero) median CO rates while the sample mean would suggest average rates.

### Gene conversion maps in *D. melanogaster*

We have detected a total of 74,453 GC events. Nevertheless, GC tracts that lay between adjacent markers are expected to be missed. Moreover, this underestimation is probably variable across the genome due to differences in SNP and marker density. Therefore, we expanded a maximum likelihood algorithm [62] that was proposed for estimating the length of GC tracts (*L_GC_*) to simultaneously estimate *L_GC_* and the rate of GC initiation (γ), and be applicable to any region of arbitrary marker distribution and density (see Materials and Methods for details).

**Figure 4:**
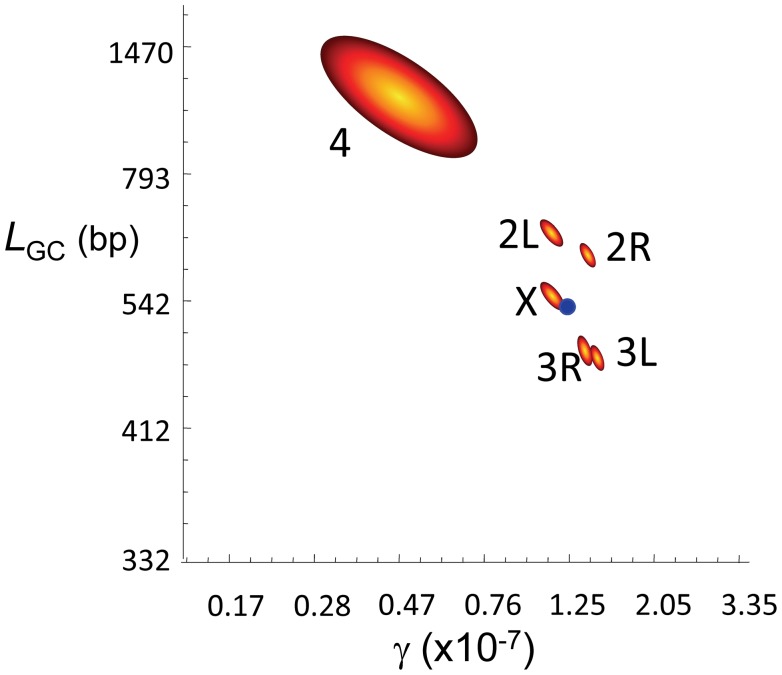
Gene conversion estimates in *D. melanogaster*. Joint maximum-likelihood estimates (MLE) of the rate of gene conversion initiation (γ) and mean gene conversion tract length (*L*_GC_) in *D. melanogaster*. γ units are per bp and female meiosis, and *L*_GC_ in bp. Red/yellow contours represent 95 confidence intervals for γ and *L*_GC_ for each chromosome arm independently. The blue dot represents the genome average for γ and *L*_GC_ based on a total of 74,453 observed GC events.

Our genome-wide estimates of γ and average *L_GC_* are 1.25×10^−7^/bp/female meiosis and 518 bp, respectively. The study of each chromosome arm separately (Figure 4) shows that arms with evidence of CO (2L, 2R, 3L, 3R and X) have similar estimates of γ (1.13–1.49×10^−7^/bp/female meiosis) and *L_GC_* (456–632 bp). Notably, we observe several GC events in the small achiasmatic chromosome fourth where CO is completely absent. Our estimates of γ and *L_GC_* for the fourth chromosome are 0.46×10^−7^/bp/female meiosis and 1062 bp, respectively.

The *rosy* locus in *D. melanogaster* is one of the best characterized in higher metazoa for intragenic recombination [63], [64]. These studies showed that GC events are more frequent than CO, with four non-crossover associated GC events to each CO [63]–[65]. In terms of absolute rate, the recovery of intragenic CO events at *rosy* reveals *c*∼3.0×10^−8^/bp/female meiosis [66] thus predicting γ∼1.2×10^−7^/bp/female meiosis at this locus. When we focus on the 100-kb genomic region encompassing the *rosy* locus our estimate of γ is 1.17×10^−7^/bp/female meiosis. At a whole-genome scale, our data suggest a γ (1.25×10^−7^/bp/female meiosis) and a ratio GC∶CO (∼83% of events result in GC) close to, albeit higher than, the estimates at *rosy*. A major difference between our results and those from the *rosy* locus however is the mean length of gene conversion tracts, with our average estimate of *L_GC_* (518 bp) significantly exceeding the estimate of 352 bp at *rosy* [62].

Another approach to estimate GC∶CO ratios is based on using an antibody to γ-His2Av as a molecular marker for DSB formation [67] and monitoring the number of γ-His2Av foci in DSB repair-defective mutants [68]. The number of estimated DSB in *D. melanogaster* using this methodology is up to 24.2 per genome [68], suggesting that 76.2% of all DSB are resolved as GC when we use the observed number of CO events per female meiosis from our data. The moderately higher fraction of GC observed in our study could be explained by differences among the strains used, if not all DSBs (or DSB-repair pathways) are marked by γ-His2Av staining [68] or if the DSB-repair defective mutants allowed for residual repair thus making some DSBs difficult to detect. Of particular interest will be future research focused on trying to localize experimentally DSBs on the fourth chromosome and other genomic regions where CO is absent but GC is detected.

**Figure 5:**
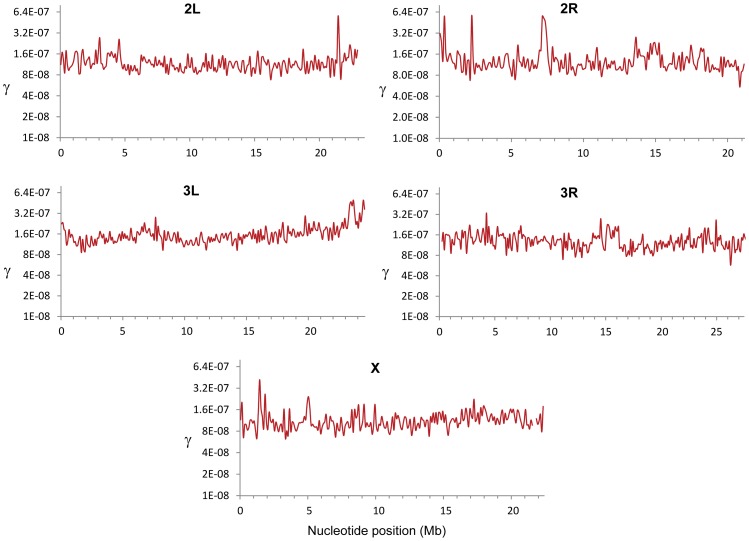
Estimates of the gene conversion initiation rate (γ) along chromosome arms in *D. melanogaster*. γ (/bp/female meiosis) based all crosses and shown for adjacent 100-kb windows.

The analysis of the distribution of γ along chromosomes at the 100-kb scale reveals a more uniform distribution than that of CO (*c*) rates, with no reduction near telomeres or centromeres (Figure 5). More than 80% of 100-kb windows show γ within a 2-fold range, a percentage that contrasts with the distribution of CO where only 26.3% of 100-kb windows along chromosomes show *c* within a 2-fold range of the chromosome average. To test specifically whether the distribution of CO events is more variable across the genome that either GC or the combination of GC and CO events (i.e., number of DSBs), we estimated the coefficient of variation (CV) along chromosomes for each of the three parameters for different window sizes and chromosome arms. In all cases (window size and chromosome arm), the CV for CO is much greater (more than 2-fold) than that for either GC or DSBs (CO+GC), while the CV for DSBs is only marginally greater than that for GC: for 100-kb windows, the average CV per chromosome arm for CO, GC and DSBs is 0.90, 0.37 and 0.38, respectively. Nevertheless, we can also rule out the possibility that the distribution of GC events or DSBs are completely random, with significant heterogeneity along each chromosome (*P*<0.0001 at all physical scales analyzed, from 100 kb to 10 Mb; see Materials and Methods for details). Not surprisingly due to the excess of GC over CO events, GC is a much better predictor of the total number of DSBs or total recombination events across the genome than CO rates, with semi-partial correlations of 0.96 for GC and 0.38 for CO to explain the overall variance in DSBs (not taking into account the fourth chromosome).

### Lack of biased gene conversion repair favoring G/C nucleotides in *D. melanogaster*

DSB resolution involves the formation of heteroduplex sequences (both for CO or GC events; Figure S1). These heteroduplex sequences can contain A(T):C(G) mismatches that are repaired randomly or favoring specific nucleotides. In a number of species, gene conversion mismatch repair has been proposed to be biased, favoring G and C nucleotides [69]–[71] and predicting a positive relationship between recombination rates (*sensu* frequency of heteroduplex formation) and the G+C content of noncoding DNA [72], [73]. In *Drosophila*, there is no direct experimental evidence supporting G+C biased gene conversion repair and evolutionary analyses have provided contradictory results when using CO rates as a proxy for heteroduplex formation ([73]–[75] but see [13], [76]). Note however that GC events are more frequent than CO events in *Drosophila* as well as in other organisms [32], [65], [77], [78] and therefore GC (γ) rates should be more relevant than CO (*c*) rates when investigating the possible consequences of heteroduplex repair.

Our data show no association of γ with G+C nucleotide composition at intergenic sequences (R = +0.036, P>0.20) or introns (*R* = −0.041, *P*>0.16). An equivalent lack of association is observed when G+C nucleotide composition is compared to *c* (*P*>0.25 for both intergenic sequences and introns). We find therefore no evidence of gene conversion bias favoring G and C nucleotides in *D. melanogaster* based on nucleotide composition. The causes for some of the previous results that inferred gene conversion bias towards G and C nucleotides in *Drosophila* may be multiple and include the use of sparse CO maps as well as incomplete genome annotation. Because gene density in *D. melanogaster* is higher in regions with non-reduced CO [13], [79], the many recently annotated transcribed regions and G+C rich exons [31], [80], [81] may have been previously analyzed as neutral sequences, particularly in these genomic regions with non-reduced CO.

### The motifs of recombination in *Drosophila*

**Figure 6:**
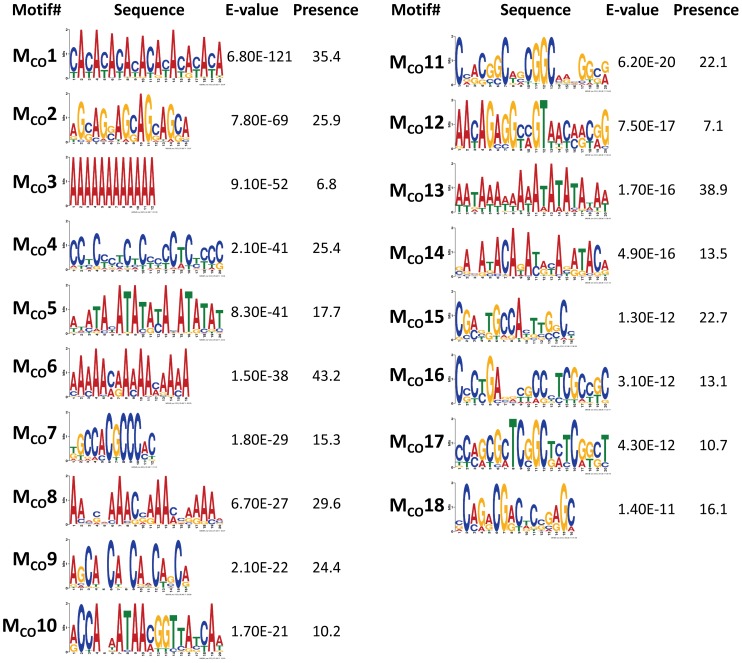
Top DNA motifs found enriched in sequences encompassing CO events. We focused on 1,909 CO events delimited by 500 bp or less (CO_500_ sequences). Only motifs with E-vale<1×10^−10^ are shown and ranked by E-value. Presence indicates the total number of motifs per 100 CO_500_ sequences, including the possible multiple presence in a single sequence. Motif M_CO_4 contains the 7-nucleotide motif CCTCCCT first associated with hotspot determination in humans [87] while motif M_CO_16 contains a 10-mer sequence (CCNTCGCCGC) that overlaps with the longer 13-mer CCNCCNTNNCCNC associated with crossover activity in human hot spots [86]. For display purposes, sequence motifs are chosen between forward and reverse to maximize the presence of A and/or C nucleotides.

**Figure 7:**
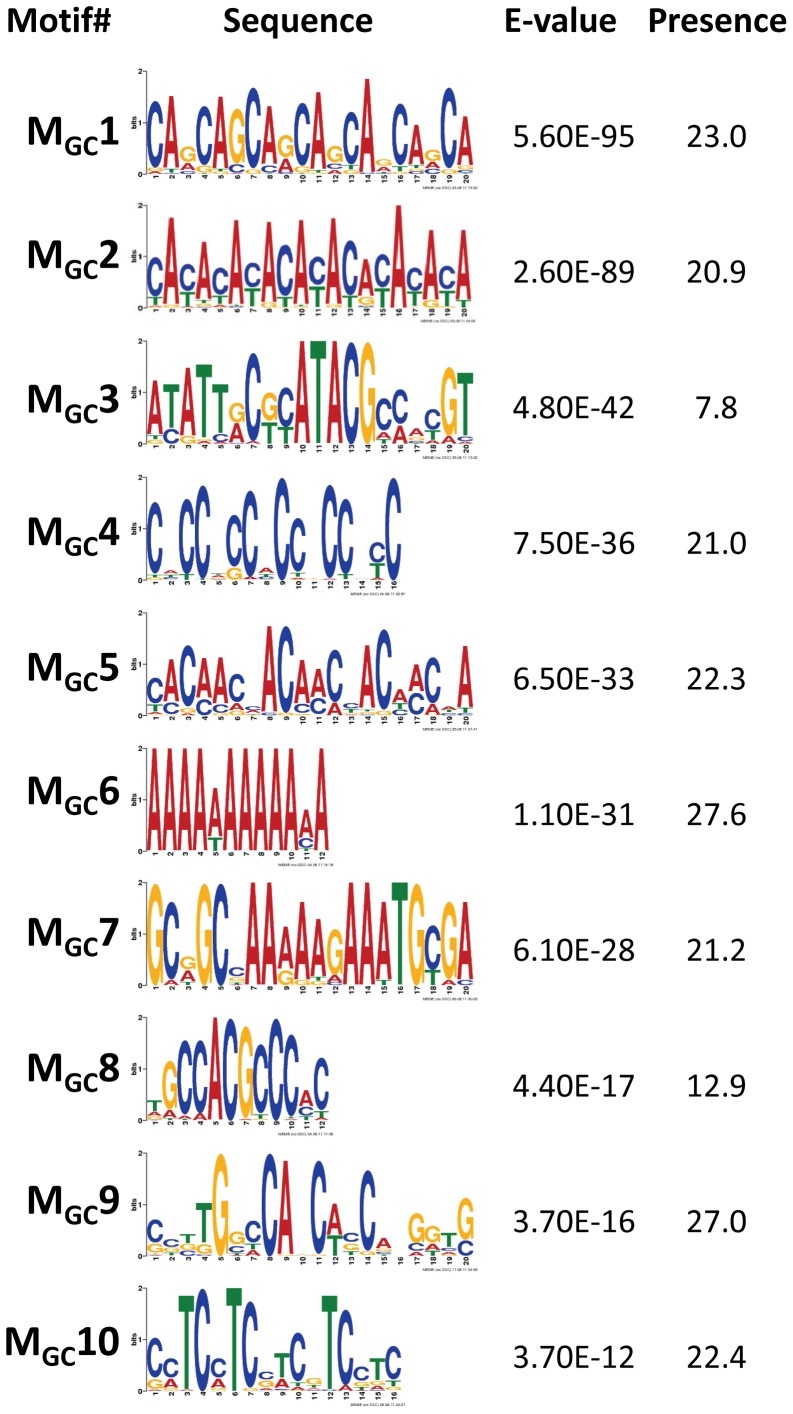
Top motifs found enriched in sequences encompassing GC events. We focused on 3,701 GC events delimited by 500 bp or less (GC_500_ sequences). Only motifs with E-value<1×10^−10^ are shown and ranked by E-value. Presence indicates the total number of motifs per 100 GC_500_ sequences, including the possible multiple presence in a single sequence. For display purposes, sequence motifs are chosen between forward and reverse to maximize the presence of A and/or C nucleotides.

To discover DNA motifs associated with recombination events (CO or GC), we focused on 1,909 CO and 3,701 GC events delimited by 500 bp or less (CO_500_ and GC_500_, respectively). Our *D. melanogaster* data reveal many motifs significantly enriched in sequences surrounding recombination events (18 and 10 motifs for CO and GC, respectively) (Figure 6 and Figure 7). Individually, the motifs surrounding CO events (M_CO_) are present in 6.8 to 43.2% of CO_500_ sequences, while motifs surrounding GC events (M_GC_) are present in 7.8 to 27.6% of GC_500_ sequences. Note that 97.7% of all CO_500_ sequences contain at least one M_CO_ motif and 85.0% of GC_500_ sequences contain one or more M_GC_ motif (Figure S4).

Previous analyses in *D. pseuddobscura* identified a significantly positive correlation between recombination rate and simple repeats, as well as the motifs CACAC [55], CCTCCCT and CCCCACCCC [29]. An equivalent study in *D. persimilis* found a positive association of CO rate with the CCNCCNTNNCCNC sequence motif known to be associated with human recombination rates [82]. A more recent study reported the motif GTGGAAA to be present near CO events in *D. melanogaster* [83]. Our study confirms the significant enrichment of some of these sequences (CACAC and CCTCCCT) and detects new ones, while there is no support for CCCCACCCC or GTGGAAA as motifs associated with increased recombination in *D. melanogaster*.

In mammals, the histone methyltransferase PRDM9 targets the 13-mer CCNCCNTNNCCNC motif described above via its zinc-finger array [84], [85], and this motif is associated with crossover activity in 41% of human hotspots [86]. None of the motifs detected in our study for either CO or GC is or contains the complete 13-mer PRDM9 motif. We do observe however shorter versions of this 13-mer within two different motifs of CO (M_CO_4 and M_CO_16 in Figure 6). Motif M_CO_4 contains the 7-nucleotide motif CCTCCCT first associated with hotspot determination in humans [87], while motif M_CO_16 contains a 10-mer sequence (CCNTCGCCGC) that overlaps with the longer PRDM9 motif.

Among the other motifs detected, we find a number of short repeats, including [CA]_n_, [CAG]_n_ and [CCN]_n_, as well as poly(A) stretches enriched in both CO_500_ and GC_500_ sequences. We ruled out the possible influence of non-LTR transposable elements and their characteristic 3′ UTR with a poly-A tail as a factor influencing our set of [A]_n_-enriched motifs, with a genomic scan showing that only 0.8% of our 500-bp long sequences used to investigate recombination motifs overlap with annotated non-LTRs. Note that the highly significantly enriched CA dinucleotide (M_CO_1 and M_GC_2) is often associated with transcription start sites (TSS) in *Drosophila* as in humans [88] and has been proposed to stimulate homologous recombination in human cells in culture [89]. Other motifs detected in this study have been also linked previously to recombination, with short poly(A) stretches associated with CO or GC in yeast [32].

Although not all motifs are shared between our sets of CO and GC events, the general picture that comes out from this study describes substantial heterogeneity in motif sequence as well as overlap between motifs for both types of recombination events. The detection of a number (or population) of motifs significantly enriched in sequences surrounding CO and GC events indicates a fundamental difference between mammalian and *Drosophila* DSB hotspots. In *Drosophila*, our results suggest DSBs occurring within larger genomic regions with high chromatin accessibility containing (or generated by) a large number of different sequence motifs.

### CO versus GC landscapes across the genome

We have detected GC events in genomic regions where CO is exceedingly rare or, as in the case of the fourth chromosome, completely absent. This result provides experimental support for a number of population genetic analyses that detected recombination events and estimated non-zero γ in these regions of the *D. melanogaster* genome [46], [90]–[95]. Our study also indicates that even across genomic regions with no apparent CO, recombination associated with GC is likely sufficient to allow for gene-specific evolutionary patterns similar to those already reported in regions with non-reduced CO (e.g., the influence of gene length and gene expression levels on codon usage bias or rates of protein evolution [12], [13], [17], [21], [96]–[99]). Based on our estimates of γ, however, we can also predict that the fourth chromosome should exhibit patterns associated with stronger linkage than other euchromatic regions with non-detectable CO.

**Figure 8:**
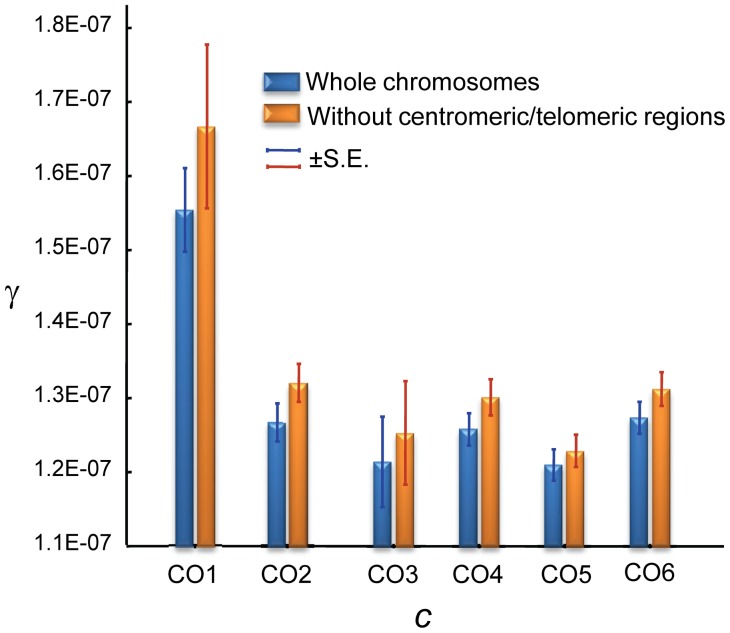
Relationship between crossing over (*c*) and gene conversion (γ) rates across the *D. melanogaster* genome. *c* and γ based on 100-kb adjacent windows with windows grouped into 6 categories of equal number according to *c* [CO1, CO2, .., CO6 indicating increasing rates of *c*] The average *c* (cM/Mb/female meiosis) values for the six categories is: 0.078 (CO1), 0.727 (CO2), 1.439 (CO3), 2.294 (CO4), 3.299 (CO5) and 5.964 (CO6). Blue columns show results when whole chromosomes are analyzed. Orange columns show results after removing centromeric and telomeric regions with visibly reduced CO rates. There is a significant negative relationship between *c* and γ using independent (non-overlapping) 100-kb windows: Spearman's *R* = −0.1246 (*P* = 1.6×10^−5^) for whole chromosomes and *R* = −0.1191 (*P* = 1.2×10^−4^) after removing telomeric/centromeric regions.

Notably, GC and CO rates are not independent. At a 100-kb scale, we observe a negative correlation between γ and *c* that is evident when analyzing whole chromosomes (Spearman *R* = −0.1246, *P* = 1.6×10^−5^,) and after removing telomeric/centromeric regions (*R* = −0.1191, *P* = 1.2×10^−4^) (Figure 8). At this physical scale the γ/*c* ratio reaches values >100 when *c*≤0.1 cM/Mb, consistent with population genetic estimates of γ/*c* at telomeric regions of the X chromosome of *D. melanogaster* [94].

Several hypotheses can be put forward to explain the different landscapes for CO and GC across the *D. melanogaster* genome. Divergent DSB repair pathways have been proposed in *Drosophila* as in yeast [100], [101], with a synthesis-dependent strand annealing (SDSA) pathway that is associated only with GC events, while the resolution of the double Holliday junction (DHJ) can generate either CO or GC (Figure S1). The detection of GC events in the fourth chromosome strongly suggests the action of SDSA, at least for a chromosome completely lacking CO, and indicates that SDSA may be acting across the whole genome. The observation that γ increases when *c* is low even after removing the fourth chromosome and telomeric/centromeric regions (see Figure 8) argues against the option of very large chromosomal domains with DSBs that are repaired by a different pathway. This same negative relationship between γ and *c*, together with the observed overlap in motifs associated with CO and GC events (see above), suggests a shared origin, and thus indicates that the disparity of landscapes for GC and CO rates across the *D. melanogaster* genome may be influenced by a difference in the relative usage of DSB repair pathways (eg., DHJ versus SDSA) or by a variable bias in the repair decision when DHJ intermediates are resolved to form either CO or GC products.

In yeast, the presence of mismatches interferes with the formation and/or extension of heteroduplex intermediates during mitotic and meiotic DSB repair [102], and sequence divergence inhibits mitotic COs to a greater extent than GCs [103]. A recent genome-wide analysis of meiotic recombination intermediates in yeast, however, suggests that mismatch repair increases the ratio CO∶GC [104]. In agreement, analyses at the *rosy* locus in *D. melanogaster* show a small increase in the ratio CO∶GC in the presence of sequence polymorphisms (27 CO and 5 GC) when compared to a case where polymorphisms are virtually absent (23 CO and 8 GC events) [66]. If this tendency is confirmed across the *Drosophila* genome and if the differences in mismatch presence across the genome are of sufficient magnitude to alter either DHJ/SDSA relative use or the resolution of DHJ into CO or GC, then genomic regions with reduced heterozygosity could favor a DSB repair favoring GC over CO events [105].

**Figure 9:**
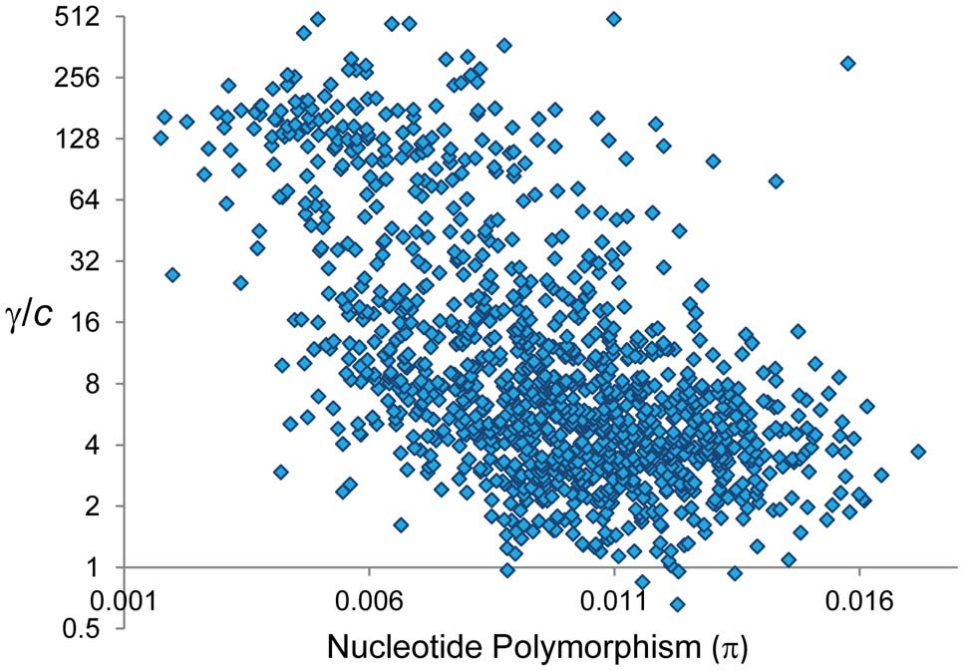
Relationship between nucleotide polymorphism (π) and the ratio γ/*c*. π indicates total pairwise nucleotide variation (/bp) based on 100-kb adjacent windows. π values for X-linked are adjusted to be comparable to autosomal regions. γ/*c* shown in log-2 scale. There is a significant negative correlation between π and γ/*c* (Spearman's *R* = −0.56, *P*<1×10^−12^) also detectable after removing telomeric/centromeric regions (*R* = −0.499, *P*<1×10^−12^).

At a whole-genome level, nucleotide differentiation between parental strains (ranging between 0.005 and 0.007 for total pairwise differences per bp) shows no association with overall γ/*c*, *c* or γ (*P*>0.4 in all cases). To test the possible influence of mismatch presence across the genome we investigated the correlation between levels of total nucleotide polymorphism (π) and the γ/*c* ratio based on adjacent 100-kb regions (Figure 9; see Materials and Methods). Congruent with the hypothesis that the choice to repair DBS into either GC or CO is heterozygosity-dependent, we observe a strong negative correlation between total π and γ/*c* across the whole genome (*R* = −0.56, *P*<1×10^−12^) and after removing telomeric/centromeric regions (*R* = −0.499, *P*<1×10^−12^). We also observe a negative relationship between total π and γ [*R* = −0.197 (*P* = 8×10^−12^) and *R* = −0.175 (*P* = 1.2×10^−8^) across the whole genome and after removing telomeric/centromeric regions, respectively]. The negative relationship between π and γ remains significant after controlling for the influence of CO rates [semi-partial correlation *r* = −0.163 (*P* = 1.2×10^−10^) across the whole genome and *r* = −0.109 (*P* = 9×10^−5^) after removing telomeric/centromeric regions].

It is also interesting to note that the observed patterns of CO and GC distribution along chromosomes can inform us about models proposed to explain chiasma interference. The “counting model” assumes that double-strand breaks occur independently, and that a fixed and organism-specific number (*m*) of noncrossovers (GC events) occur between neighboring crossovers [106], [107]. A later extension of the model included the possibility of a fraction of meiotic crossovers associated with a second pathway that is not subject to interference [108]. The extreme variation in the ratio of CO and GC events observed along chromosomes together with the negative relationship between CO and GC rates therefore seem to be inconsistent with the “counting model” while supporting a more dynamic one involving a variable DSB repair pathway or DHJ resolution across genomes.

### Local distribution of CO and GC events

**Figure 10:**
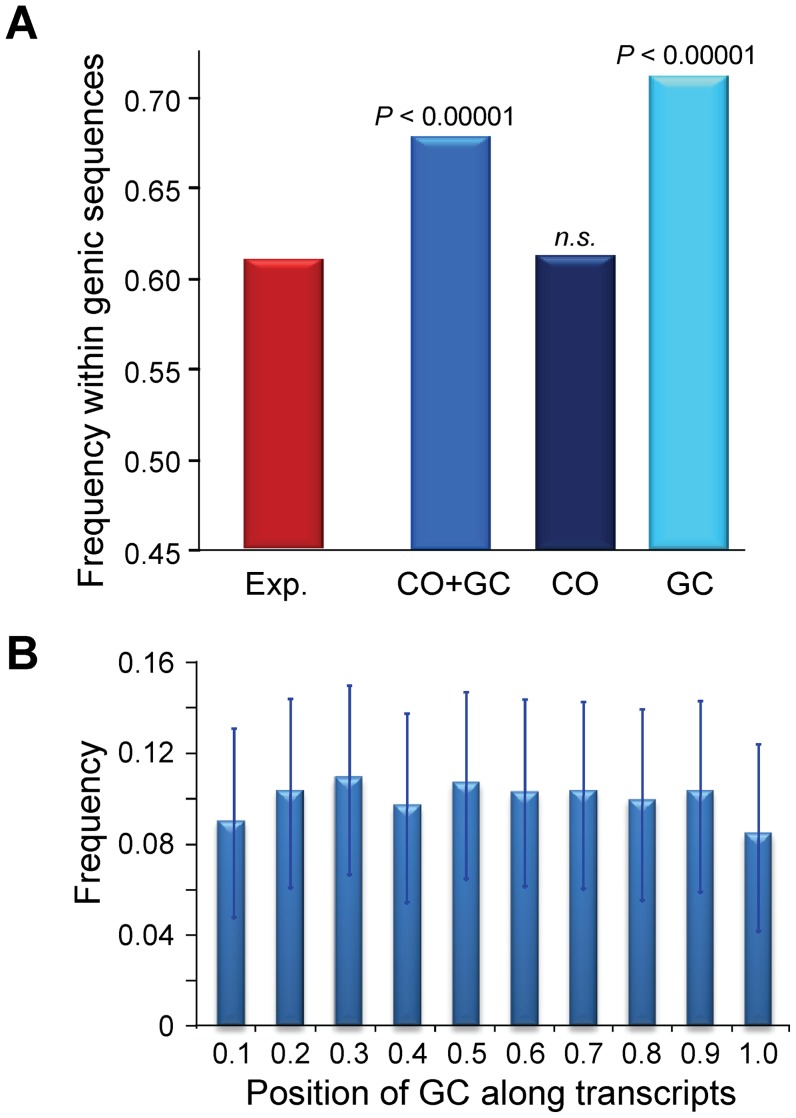
Recombination events within genic sequences in the *D. melanogaster* genome. Analyses based on 1,909 and 3,701 CO and GC events delimited by 500 bp or less (CO_500_ and GC_500_). (A) Frequency of recombination events (CO or GC) within genic sequences. Probability [*P* (Freq. Observed<Freq. Expected) based on 100,000 replicates of the observed number of recombination events distributed across the *D. melanogaster* reference genome taking into account differences in marker density between genic and intergenic regions. The expected frequency of a recombination event to be located within a genic sequence is 0.607 when taking into account the influence that maker density plays in detecting CO or GC events delimited by markers separated by 500 bp or less. The genomic location of genic sequences was obtained from the *D. melanogaster* genome annotation (release 5.3) (B) Relative position of 2,627 GC_500_ events along transcripts, shown in 10 intervals from 0 at the transcription start site (TSS) to 1 at the end of the transcript. The frequency of GC_500_ along the transcript is shown with 95% confidence intervals.

At a 100-kb scale, we have shown that CO, and to a much lesser degree GC, are not randomly distributed across chromosomes. To study the distribution of CO and GC events at a more local scale (the level of single genes) we again focused on the 5,610 CO and GC events delimited by 500 bp or less (CO_500_ and GC_500_; see above). We found that the distribution of CO and GC events is not random in terms of intergenic/genic sequences, with a significant tendency to be located within genic sequences (*P*<0.00001, Figure 10A; see Materials and Methods for details). This excess is mostly due to GC_500_, with a highly significant preference for genic regions (*P*<0.00001) while CO_500_ show no preference or avoidance (*P*>0.40). The differential distribution of GC and CO when looking at genic and intergenic sequences is consistent with the heterozygosity-dependent GC∶CO repair of DSB proposed above, given that intergenic sequences have higher levels of heterozygosity than genic sequences. Overall, our data suggest a higher probability of DSBs within annotated transcriptional units.

In yeast, some DSBs do not require transcriptional activity but depend on the binding of transcription factors, thus predicting an accumulation of recombination events near promoter regions. Alternatively, transcription may alter local chromatin structure, increasing the likelihood of DSB formation along the transcript unit ([109] and references therein). We therefore investigated the distribution of GC events along these sequences. We observe that the median position of GC_500_ is +910 from the transcription start site (TSS), close to the median midpoint of all *D. melanogaster* transcripts (+1,058). A split of transcripts into short (<2.5 kb) and long (>2.5 kb) shows the median GC_500_ position shifting significantly relative to the TSS (from +556 in short transcripts to +3588 in long transcripts; Mann-Whitney test *U* = 51,192, *P*<1×10^−12^). Moreover, the relative position of GC_500_ events along transcript sequences is uniform (Figure 10B), indicating that DSBs are not strongly associated with the binding of transcription factors. This latter result is also consistent with analyses of recombination at the *rosy* locus, where recombination is initiated throughout the gene [65]. Altogether, our results favor a model where increased chromatin accessibility contributes to the definition of DSB sites in *Drosophila*, probably associated with transcriptional processes. Note that the preponderance of GC over CO events in many species, and the difference in their physical location across the genome, may limit analyses trying to assess the role of chromatin accessibility on DSB formation and their genomic distribution when using only data associated with COs.

## Discussion

Our study provides the first coordinated high-resolution eukaryotic picture of genomic and population variation in recombination rates that distinguishes between CO and GC rates, the two outcomes of meiotic DSB repair. We report CO and GC maps that describe variation across the genome of *D. melanogaster* at the scale of 100-kb. We also obtained and analyzed eight whole-genome CO maps that provide insight into intra-specific variation at a resolution of 250-kb.

The types of recombination variation detected in *D. melanogaster* are many-fold in nature. Along chromosomes, CO rates vary significantly at the scale of 100-kb in a highly punctuated manner with hot- and coldspots within regions traditionally associated with non-reduced recombination (see also [29]). This study of *D. melanogaster* also reveals a clear difference from traditional hotspots described in humans or mice [110], [111]. Human and mice hotspots are associated with highly delimited genomic regions and a restricted number of DNA motifs [42], [86], [87], [112]. Our data suggests a softer, more probabilistic and less discrete, landscape with an excess of recombination events within larger regions (i.e., annotated transcript regions) and a large and heterogeneous population of motifs.

Our analysis of eight crosses within the same species has also allowed us to describe extensive and significant intra-specific variation in CO landscapes. The presence of polymorphic modifiers is at the core of models of the evolution of recombination ([113] and references therein) but little is known about their actual frequency and genomic distribution in natural populations. Our collection of maps in *D. melanogaster* describing naturally occurring variation in recombination rates underscore the tremendous potential for selection to act on or be associated with recombination modifiers, and alter the landscape of recombination rates across the genome.

Notably, the excess variance in recombination among crosses is mostly associated with CO hotspots at low frequency in our sample. Our results therefore emphasize the need for obtaining estimates of recombination based on several crosses rather than on a single or two crosses, even if highly detailed in resolution. Additionally, the presence of hotspots at low frequency suggests that estimates of recombination rate based on the average of several maps may not fully capture the relevant population dynamics either under neutrality or as a consequence of the interaction between selection and linkage.

The observed pattern of hotspots at low frequency could be explained by mutation-selection balance but is also is congruent with neutral expectations where most derived mutations, in this case modifiers causing high recombination at specific genomic regions, are expected to be at low frequency (present as singletons or in two genotypes in our sample). For *cis*-controlled hotspots, their frequency might be also limited by the meiotic drive predicted when the initiating DSB sequence is converted into the allele of the non-initiating homologous chromosome during DSB repair [114]–[119]. Only a systematic study of populations and closely related species will allow us to assess the relative role of selection, mutation and drift acting on these modifiers.

**Figure 11:**
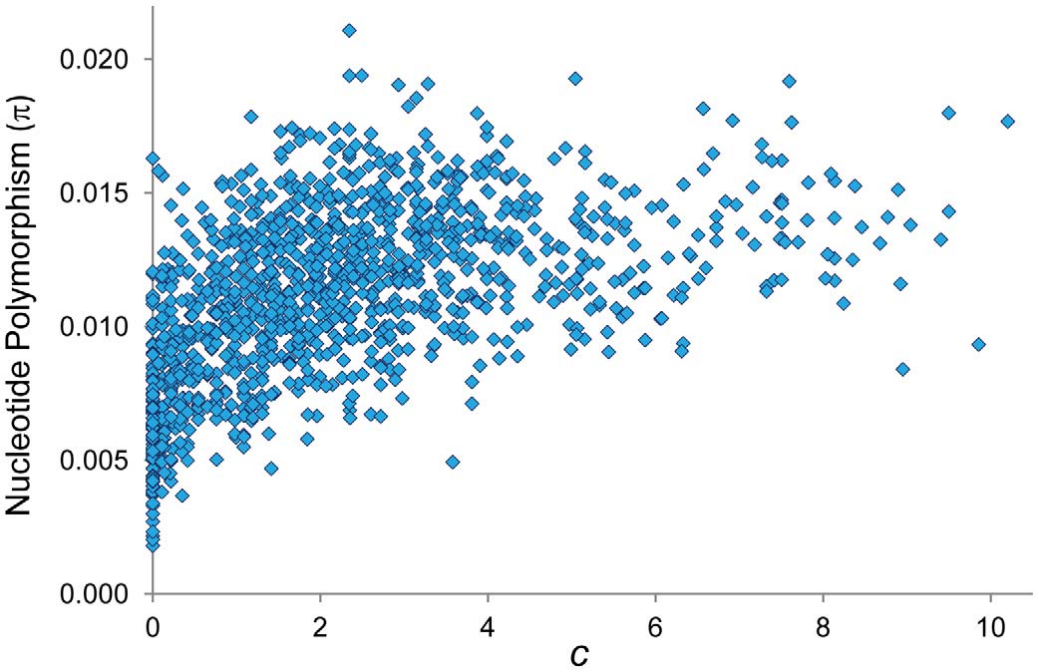
Relationship between CO rate (c) and nucleotide polymorphism (π). π indicates pairwise nucleotide variation (/bp) at noncoding sites (intergenic and introns). π values for X-linked are adjusted to be comparable to autosomal regions. Based on 100-kb adjacent windows, there is a significant positive correlation between *c* and π (Spearman's *R* = 0.560, *P*<1×10^−12^) also detected after removing telomeric/centromeric regions (*R* = 0.497, *P*<1×10^−12^).

The existence of coldspots throughout the whole genome, monomorphic or coupled with low median values, has also direct implications for population genetic analyses of selection. A reduction in polymorphism levels in regions of non-reduced recombination is often explained by the action of positive selection and selective sweeps (the hitchhiking model, HH [120]–[124]). Similar population genetic patterns however could be explained by the steady elimination of deleterious mutations from populations (the background selection model, BGS [48], [125], [126]) if sufficiently reduced CO rates were operating. The use of our high-resolution CO maps (*c*) to investigate the predicted consequences of selection at linked sites (HH and/or BGS) across the *D. melanogaster* genome confirms the results of previous analyses [10], [13], [19], [21], [22], [127]–[130] and show a positive relationship between *c* and levels of nucleotide polymorphism (π) at noncoding sites (Figure 11; *R* = 0.560, *P*<1×10^−12^). Notably, our CO maps reveal that the strong association between *c* and π is also observed after removing telomeric/centromeric regions (*R* = 0.497, *P*<1×10^−12^).

Our results therefore suggest that BGS should be considered across the whole *D. melanogaster* genome before invoking positive selection and selective sweeps. In this regard, strong local BGS caused by the presence of CO coldspots embedded in region of non-reduced recombination (what we call “*coldspot effect*”) would predict the presence of genomic regions with polymorphism and divergence patterns (e.g., reduced neutral polymorphism and an excess of amino acid substitutions) that could be also interpreted as evidence of adaptive evolution [131].

We observe that GC rates are more uniformly distributed than CO rates and are also detectable in regions with reduced or absent CO. Because GC is predicted to play a more important role in increasing *effective* recombination at short distances than CO [45], [48], [105], this result is specifically relevant for studies of variation in evolutionary patterns at physically close regions. Nested within large-scale recombination environments, total recombination and GC events in particular tend to occur within annotated transcript sequences. Our data therefore expose higher recombination within transcript regions relative to intergenic regions. Introns and/or synonymous sites thus might not be fully adequate to test evolutionary patterns at flanking regions of these same genes, and vice versa. For instance, if we accept the presence of conserved sites in transcribed regions (e.g., exons) and flanking regions (e.g., cis-regulatory sequences), local BGS together with variable effective recombination might generate patterns of lower polymorphism (and polymorphism to divergence) in noncoding flanking regions relative to intronic or synonymous sites.

Current population genetic analyses use coalescent simulations to test alternatives to a neutral process or specific models of selection [132]–[138]. These coalescent simulations can use constant recombination rates (*c* and/or γ) or add flexibility by including variable rates between simulations, but assume nonetheless that recombination occurs with equal probability for different lineages or for adaptive and neutral alleles within a genealogy [139], [140]. Our data indicate that coalescent-based analyses should take into account the highly heterogeneous recombination environments at long- and local-scales across genomes but also incorporate recombination variation *within* genealogies in order to capture the dynamics associated with naturally occurring modifiers.

An additional and intriguing possibility for models of selection and linkage is the support that our results provide for a heterozygosity-dependent GC∶CO repair of DSB in *Drosophila* (see also [46]), increasing the frequency of GC relative to CO events when heterozygosity is low. Under this scenario, selection would reduce nucleotide polymorphism at linked sites due to BGS or HH but this very same change in polymorphism levels would, in turn, alter the degree of future linkage effects due to the variable GC∶CO ratio [46]. Further studies are needed to understand and characterize the spatial and temporal dynamics across the genome generated under this model, with variable GC∶CO ratios and stochastic and/or selective processes associated with polymorphic modifiers of DSB formation.

In conclusion, we have shown that there are many layers of variation in recombination rates in *D. melanogaster*: across genomes at a large- (>100-kb) and small- (i.e., single-gene) scales, among crosses and for GC and CO. Each of these levels is expected to play a role in shaping evolutionary patterns. We propose that these overlapping, non-independent and dynamic landscapes of recombination should be included in a new generation of population genetic models of selection and recombination.

## Materials and Methods

### *Drosophila melanogaster* strains

We generated eight crosses using 12 highly inbred strains of *D. melanogaster*. Ten of these strains were recently generated by the laboratory of Trudy Mackay from a natural population collected in Raleigh (NC, USA) after 20 generations of full sib mating (RAL strains). Importantly, these strains have maintained substantial genetic/phenotypic variation between lines [141], [142]. The RAL strains used in this project are: RAL-208, -301, -306, -375, -380, - 391, -514, -712, -786 and -820. We also used two non-US strains, collected in Madagascar (MDG) and Papua New Guinea (PNG) obtained from the Bloomington *Drosophila* Stock Center (Indiana University). Table 1 indicates the eight crosses under study. Our stocks of RAL-786 and -820 carried fixed inversions for chromosome arm 3R and therefore the analyses of recombination rates for this chromosome arm excluded these two crosses.

**Table 1:** Cross, parental strains, and number of female meioses.

Cross #	Parental Strain 1	Parental Strain 2	Number of female meioses
1	PNG	MDG	762.5
2	RAL-208	RAL-375	1522
3	RAL-306	RAL-391	493.5
4	RAL-375	RAL-514	720
5	RAL-208	PNG	752.5
6	RAL-301	RAL-375	627
7	RAL-712	RAL-786	499.5
8	RAL-380	RAL-820	483

The genomes of the RAL strains have been sequenced [The *Drosophila* Population Genomics Project (DPGP [17]), and The *Drosophila* Genetic reference Panel (DGRP [142]). Nevertheless, and for all strains including RALs, we obtained Illumina sequence reads and generated genomic sequences of the strains used in our laboratory for crosses to get an accurate (current) description of SNPs and small indels for all parental strains, including the possible presence of heterozygous sites.

### Crosses and generation of Recombinant Advanced Intercross Lines (RAILs)

A number of factors can influence recombination in *D. melanogaster* including female age, temperature, number of matings or food [57]–[60]. To reduce the effects of these factors, all crosses and strains were maintained at the same temperature (23C) under a 12 hr. light/dark cycle, with constant fly density in half pint bottles on standard corn-meal media. Recombination is higher for young (1–2 days) and old (>12 days) females [59]. Therefore all crosses were carried out with flies of equivalent age, with 6 hour old virgin flies allowed to mate and lay eggs for a period of 36–48 hours, after which the parents were discarded and the offspring were allowed to emerge. For all crosses, parental strains were crossed in both directions to average out possible maternal effects on recombination rates.

Standard approaches to assess rates of crossing over often cross two inbred strains and genotype progeny from F2 backcrosses. To maximize the number of recombination events per fly genotyped, we generated recombinant advanced intercross lines (RAIL). To generate RAILs by sibling mating and minimize homozygosity, we crossed five groups of forty F_1_ flies, twenty males and twenty females, randomly collected from the previous generation and put together in new bottles. The F_1_ flies were also allowed to mate for 1.5–2 days after which they were discarded and F_2_ offspring were allowed to emerge. To generate the next generation, flies from all bottles were randomly mixed to create the next groups of twenty males and twenty females. For all crosses, the number of bottles was increased progressively, starting with five up to ten, to reduce the effect of random genetic drift that could generate homozygous combinations for recombination events thus underestimating recombination rates.

Our crosses varied in terms of sib-mating generations, ranging between 1 (standard approach with a single meiosis in heterozygous *D. melanogaster* females) and 10 generations. To estimate the number of female meioses associated with each cross we modeled our crossing design with simulations mimicking the corresponding mating process, including the number of individuals (males and females) and bottles per generation (see Table 1 for the number of meioses corresponding to each cross). The use of the ‘equivalent meioses’ estimated with this approach generated a genetic map length for *D. melanogaster* of 287.3 cM. This value closely matches classical measures (282 cM) in this species [26] thus indicating that our approach to estimate the number of meiosis captures appropriately the map expansion with number of generations and the crossing design. Table 2 shows the genetic map length for each cross and chromosome arm.

**Table 2:** Genetic map length per chromosome arm and cross.

Cross #	X	2L	2R	3L	3R
1	71.8	49.8	45.8	54.9	47.6
2	66.2	55.8	53.8	52.7	60.8
3	56.4	64.9	60.0	49.9	56.7
4	63.4	62.5	51.4	51.8	61.3
5	67.5	55.6	49.0	55.9	56.2
6	66.6	52.4	55.0	50.3	66.5
7	73.0	55.9	58.6	53.4	*
8	57.7	57.5	59.3	53.9	*

* Fly stocks RAL-786 and -820 carry fixed inversions for chromosome arm 3R.

Finally, female *D. melanogaster* RAILs were crossed to *D. simulans* males to obtain hybrid females for genotyping. We originally used *D. simulans* males from strain *w501*, but we finally decided to use *D. simulans* males from the strain Florida City due to the much higher fraction of hybrids produced. *D. melanogaster×D. simulans* crosses were conducted using *D. simulans* males aged for 3–4 days before combining them for interspecific mating. After mating, *D. simulans* males were discarded and *D. melanogaster* females were individualized and maintained in vials to lay eggs. For each cross between two parental strains, we froze (−20°C) the hybrid offspring of more than 350 mated *D. melanogaster* RAIL females. To ensure replicates and resequencing if needed, we froze 6 hybrid offspring females from each *D. melanogaster* mated females. Figure S5 shows a schematic representation of the crossing methodology. Note that hybrids are used for genotyping only and the meiotic products from these hybrids are not analyzed. In total, we sequenced 2,829 hybrid females (with 2,829 single-fly Illumina libraries, see below), corresponding to 5,860 meioses.

### DNA extraction

We extracted DNA from single hybrid females using Tissue Lyser LT (Qiagen, Valencia, CA) followed by a modified Qiagen DNeasy Blood & Tissue Kit (Qiagen, Valencia, CA) protocol. 90% of the eluted DNA was used for preparing a library for each individual fly. The rest of the sample was frozen and kept for posterior validation of markers, crossing over (CO) or gene conversion (GC) events if needed. The DNA of each fly was fragmented using a Bioruptor UCD-200 (Diagenode, Denville, NJ) with 0.5 ml tubes and settings that maximize the concentration of sheared DNA at 300 bp. Most of the eluted fragmented DNA was used for library preparation while the remaining (15%) of the sample was used for analysis of fragmented DNA for each individual fly by measuring the relative amount of a segment of the gene *rp49* using Real-time PCR analysis (Roche LightCycler 480; Roche, IN, USA). This first quantification of single fly DNA served to confirm successful DNA extraction to be used for Illumina libraries. A second relative quantification of DNA was also performed after ligation of Illumina adapters (see below) following an equivalent protocol and used to normalize the amount of library DNA from each fly for multiplex sequencing.

### Illumina library preparation

Single fly Illumina libraries of sheared DNA were prepared using NEBNext reagents and protocols (New England Biolabs, MA, USA) scaled-down appropriately for small amounts of DNA (detailed protocol available from J.M.C. upon request) and using indexed Illumina adapters. When sequencing multiplexed samples (using different tags) there is the possibility of mistakenly assigning reads to the wrong sample due to sequencing errors in the tag sequence. The likelihood of miss-assignment rapidly increases with, 1) shorter tags, 2) with the use of sequence reads not perfectly matching tag sequences and, 3) when tag sequences are only one or two nucleotides away from other tag-sequences. To reduce this potential error as much as possible our custom 7-nucleotide tags (7 nt plus the required ‘T’ for Illumina primer ligation) were designed to be a minimum of 4 nucleotide changes away from any other tag. Additionally, we only used Illumina reads with initial sequences completely identical to the tags used. This conservative approach is needed to prevent read miss-assignment in marker-based, light sequencing studies. Our 24 custom-designed 7-nucleotides tags are described in Table S1. The use of 24 indexed Illumina adapters allowed us to multiplex 24 single-fly libraries in a single Illumina lane to be later separated computationally.

The PCR enriched libraries were validated by running an aliquot on a standard agarose gel. Final concentration quantification was obtained with Quant-iT PicoGreen dsDNA Reagent and Kits (Invitrogen, CA, USA) on a Turner BioSystems TBS-380 Fluorometer. Twenty four equimolar PCR-enriched libraries were pooled together to obtain a final set of multiplexed single-fly libraries that would be sequenced in a single Illumina lane. Sequencing was mostly performed using the Illumina Genome Analyzer IIx and Illumina HiSeq 2000 instruments at the Iowa State University DNA Facility with additional plates sequenced at the High Throughput Sequencing Facility of the University of North Carolina at Chapel Hill using the Illumina Genome Analyzer IIx.

### Genomic analyses and bioinformatic pipeline

#### Generation and annotation of parental reference sequences

We generated reference genomic sequences for all *D. melanogaster* parental strains and for the *D. simulans* Florida City strain that we used for our crosses. We obtained 75- and 100-nt Illumina single reads in excess of 25-fold coverage for the *D. melanogaster* RAL strains and in excess of 40-fold coverage for *D. melanogaster* MDG and PNG and for *D. simulans* Florida City strains. To generate RAL reference parental sequences we added the sequencing reads from the NCBI short read archive (SRA) study SRP000694 [142] to our 75-nt or 100-nt reads. Filtering of reads, mapping and generation of consensus reference sequences for parental strains was carried out using the FASTX-toolkit (http://hannonlab.cshl.edu/fastx_toolkit/), BWA [143], SAMtools v1.4 [144] and a collection of custom PERL scripts using as reference the *D. melanogaster* genome sequence (r.5.30; http://flybase.org).

Contrary to standard approaches to generating consensus sequences based on SNP calling, we generated parental reference sequences specifically intended for our mapping purposes. We focused on taking into account heterozygous sites in parental strains that could miss-assign the origin of individual reads as well as annotate as unreliable sites those sites with limited representation (coverage). Two distinct issues associated with heterozygosity within strains were detected. First, residual heterozygosity (present when the lines were originally sequenced, ca. 2008–2009) and maintained in the strain that was used in our laboratory for crosses. Second, sites showing a different high-frequency/monomorphic variant in our laboratory relative to when they were originally sequenced.

We annotated (marked) each potential heterozygous site in the reference sequence of parental strains as ambiguous sites using the appropriate IUPAC ambiguity code using a permissive approach. We used full (raw) pileup files and conservatively considered as heterozygous site any site with a second (non-major) nucleotide at a frequency higher than 5% regardless of consensus and SNP quality. For instance, if the sequencing of a parental strain of *D. melanogaster* generates 12 reads exhibiting an ‘A’ and 1 read exhibiting a ‘G’ at a particular nucleotide position, the reference will be marked as ‘R’ even if consensus and SNP qualities are 60 and 0, respectively. We assigned ‘N’ to all nucleotide positions with coverage less that 7 regardless of consensus quality because of the lack of information on their heterozygous nature. We also assigned ‘N’ to positions with more than 2 nucleotides.

This approach is conservative when used for marker assignment because the mapping protocol (see below) will remove heterozygous sites from the list of informative sites/markers while also introducing a “trapping” step for Illumina sequencing errors that may be not fully random. Finally we introduced insertions and deletions for each parental reference sequence based on raw pileup files.

#### Mapping of reads and generation of *D. melanogaster* recombinant haplotypes

Sequences were first pre-processed and only reads with sequences exact to one of tags were used for posterior filtering and mapping. FASTQ reads were quality filtered and 3′ trimmed, retaining reads with at least 80% percent of bases above quality score of 30, 3′ trimmed with minimum quality score of 12 and a minimum of 40 bases in length. Any read with one or more ‘N’ was also discarded. This conservative filtering approach removed an average of 22% of reads (between 15 and 35% for different lanes and Illumina platforms).

We then eliminated all reads with possible *D. simulans* Florida City origin, either truly originating from the *D. simulans* chromosomes or with *D. melanogaster* origin but similar to a *D. simulans* sequence. We used MOSAIK assembler (http://bioinformatics.bc.edu/marthlab/Mosaik) to map reads to our marked *D. simulans* Florida City reference sequence. Contrary to other aligners, MOSAIK can take full advantage of the set of IUPAC ambiguity codes during alignment and for our purposes this allows the mapping and removal of reads when represent a sequence matching a minor allele within a strain. Moreover, MOSAIK was used to map reads to our marked *D. simulans* Florida City sequences allowing 4 nucleotide differences and gaps to remove *D. simulans* -like reads even with sequencing errors. We further eliminated *D. simulans* -like sequences by mapping remaining reads to all available *D. simulans* genomes and large contig sequences [*Drosophila* Population Genomics Project; DPGP, http://www.dpgp.org/] using the program BWA and allowing 3% mismatches. The additional *D. simulans* sequences were obtained from the DPGP website http://www.dpgp.org/ and included the genomes of six *D. simulans* strains [w501, C167, MD106, MD199, NC48 and sim4+6; [17]] as well as contigs not mapped to chromosomal locations.

After removing reads potentially from *D. simulans* we wanted to obtain a set of reads that mapped to one parental strain and not to the other (informative reads). We first generated a set of reads that mapped to at least one of the parental reference sequences with zero mismatches and no indels. At this point we split the analyses into the different chromosome arms. To obtain informative reads for a chromosome we removed all reads that mapped to our marked sequences from any other chromosome arm in *D. melanogaster*, using MOSAIK to map to our marked reference sequences (the strain used in the cross as well as from any other sequenced parental strain) and using BWA to map to the *D. melanogaster* reference genome. We then obtained the set of reads that uniquely map to only one *D. melanogaster* parental strain with zero mismatches to the marked reference sequence of the chromosome arm under study in one parental strain but not in the other, and vice versa, using MOSAIK. Reads that could be miss-assigned due to residual heterozygosity or systematic Illumina errors would be removed in this step.

Before considering the remaining reads as informative markers we incorporated additional filtering steps. We removed all reads that would differentially map to one parental reference sequence and not to the other if one of the reference sequences corresponding to this read contained one or more ‘N’s. Although the dominant error type in Illumina sequencing is substitutions (as opposed to indels), indels are more difficult to deal with and to incorporate uncertainty and therefore we removed reads that would differentially map to one parental reference sequence and not to the other if the parental reference sequences at this region differ by an indel. We also removed reads that showed nucleotide differences between parental strains at the 5′ or 3′-end of the read. Finally, we considered informative reads only those that distinguish between parental reference sequences with a maximum of 3 single-nucleotide, non-consecutive differences. The informative reads from each genotyped hybrid female were used to generate *D. melanogaster* (possibly recombinant) haplotypes for each chromosome arm.

### Detection of CO and GC events

We mapped CO and GC events directly to each individual *D. melanogaster* haplotype (from a single RAIL hybrid) and not based on the combined analysis of all *D. melanogaster* haplotypes for a given chromosomal region. That is, most chromosomes only show 1 to 4 (in the case of several generations of RAIL) CO events. Due to the elevated density of markers each CO is supported by numerous (hundreds and often thousands) contiguous markers at either side and therefore we expect to have detected all COs. GC events on the other hand are supported by single or a few adjacent markers that do not extend over long stretches of DNA (i.e., much shorter than 25 kb).

In principle, double CO in a single meiosis (or two independent COs in different meiosis) could be mistaken for long GC if they were very close to each other. GC events are assumed to be short, often shorter than 500 bp and be exceptionally rare above 10–15 kb [62]. We analyzed marker maps to locate CO and GC events along single chromosomes, using a cut-off for maximum tract length for GC (*L_GC_*) of 15 kb. Several lines of evidence suggest that this approach classifies correctly CO and GC events based on our experimental design. First, equivalent maps for CO and GC were obtained when applying a cut-off of 25 kb, suggesting that at 15 kb we are classifying as GC most if not all detectable GC events and that, when two or more CO events occur in the same chromosome in our RAILs, these COs are separated by more than 25 kb. Second, crosses involving several generations of RAIL show equivalent number of CO per chromosome per female meiosis to crosses based on a single meiosis. Finally, simulations of CO distribution along chromosomes following the mating protocol used to generate RAILs, with a cut-off of 15 kb to assign GC and the conservative assumption of no CO interference shows a maximum erroneous assignment of 0.16% and 1.4% assuming random distribution or the observed distribution of CO, respectively.

We detected a total of 32,511 CO events and CO maps for each cross and chromosome arm were generated by directly combining the observed COs from all individual haplotypes and tabulated along each chromosome in terms of *c* [centimorgans (cM) per megabase (Mb) per female meiosis].

### Estimates of gene conversion initiation rates (γ) and GC tract length (*L_GC_*)

Our study revealed a total of 74,453 GC events. Nevertheless, a fraction of GC events are expected to be missed due to GC tracts that lay between adjacent markers. Moreover, this underestimation is predicted to be variable across the genome due to differences in SNP and marker density. Our data consists of a great many independent GC events distributed across different haplotypes for a given chromosome, each GC event likely defined by different SNPs and a different distance from adjacent SNPs. The nature of this dataset differs from previous population genetic studies of gene conversion [94], [145] as well as from experimental studies that based their results on genetic crosses that directly detected presence/absence of GC events using a limited number of informative markers and/or focused on a specific genomic region [62], [112]. SNPs not involved in GC events, each separated by a different distance from adjacent SNPs, are also informative about the rate of GC initiation (γ) and length of GC tracts (*L_GC_*).

We therefore expanded a previous maximum likelihood algorithm [62] to estimate *simultaneously* γ and *L_GC_* and to be applicable to any region of arbitrary size with variable density SNP/marker data that takes into account both observed GC events and markers not involved in GC events. Each observed, unselected, GC tract will be treated as a different event defined by the outmost markers (left and right nucleotides) of the *observed* tract that describe the minimum true tract length (*L*_min_; *L*_min_≥1). We also know that a tract has a left end and a right end delimited by the nearest left/right flanking markers not involved in the tract, with *m_gc_* indicating the average number of nucleotides between the observed tract and the left and right flanking markers. The maximum tract length (*L*_max_) is then *L*_min_+2(*m_gc_*).

Following Hilliker et al. (1994) [62], gene conversion tract lengths can be described by a geometric distribution that assumes independence of each nucleotide-adding step with a probability *φ*. The probability of a GC tract of length *n* nucleotides can be described by
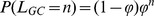
with the mean tract length
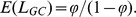
The likelihood of an observed GC event that encompasses the observed tract is then
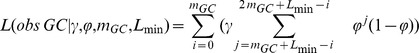


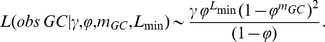



Markers not involved in GC tracts either due to no GC event or because GC tracts initiate and terminate between two 2 markers are also informative. These markers are separated by *m* nucleotides and we preserve the possibility that *m* differs from *m_gc_*. Let 1- *φ^n^* denote the probability of a GC tract shorter than *n* nucleotides. Then
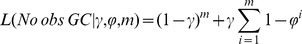


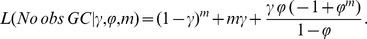



For a complete dataset with *k* GC events and *t* markers not being involved in GC events, the total Likelihood of the data is
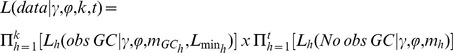
or its log for convenience. Finally we can obtain numerically the Maximum Likelihood Estimate (MLE) of γ and *L_GC_* using the log-likelihood function for our dataset(s). We have applied this approach to estimate γ and length *L_GC_* for the whole genome as well as for each and along chromosome arms.

### Validation

#### *In silico* False Discovery Rate (FDR) analysis

Although we have strived for designing a protocol that includes a hefty number of filters and mapping controls, we anticipate a non-zero rate of misplacing reads given the massive number of reads obtained per cross. We estimated our false discovery rate (FDR) for CO and GC events by generating random collections of Illumina reads when there is no expectation of detecting any recombination (CO or GC) event. We applied the same bioinformatic pipeline used to identify informative markers, generate *D. melanogaster* haplotypes and ultimately identify CO and GC events and estimate *c* and γ.

We investigated the efficacy of our filtering/mapping protocol by generating collections of reads with 50% of reads from a single parental *D. melanogaster* (eg, RAL-208) and 50% of reads from the *D. simulans* strain used in all crosses (Florida City) to closely represent the reads from a single hybrid female fly when there is no expectation for any CO or GC event. The reads used for this study were obtained from our Illumina sequencing effort of parental *D. melanogaster* and the *D. simulans* strains used in this study (see above) and were used with no a priori knowledge of their sequence and mapping quality, Each *in silico* library is, on average, equivalent to individual hybrid libraries in terms of number of reads with the only difference that we removed the first 8 nucleotides of each read from the parental lines (equivalent to the removal of the 5′ (7 nt+‘T’) tag in our multiplexed hybrid reads). This approach to estimate FDR takes into account possible limitations in the filtering and mapping algorithms and protocols, Illumina sequencing errors (random and non-random), the effects of non-complete or inaccurate reference sequences and the bioinformatic pipeline.

We generated 400 *in silico* random library collections (the average number of libraries per cross), applied the same bioinformatic pipeline and parameters used for the filtering and mapping of reads from our crosses and estimated CO and GC rates. Because the expectation is zero for both CO and GC we can compare these rates to those from actual crosses to obtain a suitable FDR. Our results show that no CO event would be inferred when using only one *D. melanogaster* parental strain and *D.simulans* (zero events in all 400 in silico libraries compared to the more than 2,000 detected per cross). GC events are however detected. Overall, we can infer that 4.1% of our inferred GC events can be explained by miss-assigned reads and that most of these erroneously mapped reads are from the *D. melanogaster* strain, not from the parental *D.simulans*. This FDR varies among chromosomes, highest and lowest for the 3R (6.2%) and X (1.9%) chromosome arms, respectively. Zero GC events (in 400 *in silico* libraries) were inferred in the small chromosome 4.

Worthy of note, preliminary FDR studies based on more straightforward filtering/mapping protocols revealed higher percentages of miss-assigned reads that would overestimate the number of GC events. These results guided us to include additional steps to our bioinformatic pipeline. The additional steps included in our final protocols (see above) consisted in, 1) increased filtering stringency of reads before mapping, 2) use of additional *D. simulans* sequences and contigs (additional to the Florida City reference) for ‘trapping’ purposes of *D. simulans* -like sequences, 3) removal of reads mapping to chromosomes other than the one under study (with permissive mapping allowing up to 10% mismatch), and 4) removal of reads that differentiate parental sequences if these sequences differ by an indel.

#### Experimental PCR/sequencing of gene conversion events

When we extracted DNA from single hybrid females we froze ∼10% of the total DNA for posterior validation of markers, crossing over (CO) or gene conversion (GC) events if needed. We designed primers, PCR amplified and sequenced the genomic regions surrounding 31 GC events detected based on our bioinformatic pipeline. These 31 GC events were chosen to represent GC events assessed based on a single informative marker, in the fourth chromosome or with long GC tract (>2 kb). 30 out of these 31 showed SNPs patterns confirming the presence of a GC event. Five of these confirmed GC events were located in the fourth chromosome. Overall the GC events ranged in tract length, from 171 bp to a maximum of 9,585, the latter located in the X chromosome (ca. position 2.6 Mb).

### Estimates of recombination heterogeneity along chromosomes

Heterogeneity in recombination events (CO or GC) along chromosome arms was investigated as the probability of observing equal or greater coefficient of variation (CV) among non-overlapping regions. The size of the genomic regions ranged from 100-kb up to half the chromosome length (10 Mb) in 100-kb increments. Probabilities were obtained based on 10,000 replicates per window size and chromosome, and assuming a random distribution of events. Heterogeneity was investigated for whole chromosomes and after removing centromeric and telomeric regions with visibly reduced CO rates (see below).

### Estimates of variation in recombination maps among crosses

We studied intra-specific variation in CO rates by estimating the variance to mean ratio, or Index of Dispersion (*R*), and tested whether the observed variance in CO events among crosses can be explained by a Poisson process. This approach assumes that for a given chromosomal region the number of observed CO events in different crosses is a set of independent and identically distributed random variables. In the case of Poisson distributed data, the expectation is *R*∼1.

To focus our study on variation in the *distribution* of CO rates and take into account genome-wide differences between crosses and the different number of meiosis analyzed, we used weighting factors (*w*) that include the total number of CO per chromosome in a particular cross as ‘lineage effects’. The weighting factor of cross *i* for a given chromosome (*w_i_*) is then
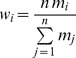
with *m_i_* representing the total number of CO events in a chromosome in cross *i*, and *n* the number of crosses analyzed (in our case *n* = 8). Following Gillespie (1989) [146], we can obtain the index of dispersion for CO events (*R*_CO_) for region *r* based on
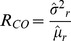
with
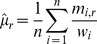


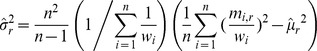
where 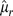, 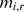 and 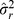 indicate the mean number of recombination events at region *r*, the number of CO events in region *r* for cross *i*, and the unbiased estimator of the variance at region *r*, respectively.

Assuming a Poisson distribution of CO events, *R*_CO_ values significantly greater than 1 would indicate an excess variance (overdispersion) of CO events among crosses for the region under study. We estimated the statistical significance of *R*_CO_ values being greater than expected using simulations that assume a Poisson-distributed total number of CO events that are randomly distributed among crosses (once corrected for lineage effects) and along chromosomes. Note that the variance due to binomial sampling is greater than that for Poisson, thus the expectation for binomial distributed data is *R*<1, making our approach conservative when assessing overdispersion of CO events among crosses. Probabilities were obtained based on 100,000 independent replicates. *R*_CO_ along chromosomes was investigated for non-overlapping regions with sizes ranging from 250 kb to 1 Mb.

### Local distribution of CO and GC events

Because we focused on CO and GC events delimited by 500 bp or less (CO_500_ and GC_500_), heterogeneity in the density of informative markers across the genome could bias our expected, null, distribution of CO_500_ and GC_500_ events. Indeed, informative markers are 1.4% more likely to be located in intergenic regions than within genic regions. We then used this “marker-density corrected” approach to obtain the expected frequency of CO_500_ and GC_500_ recombination events within genic sequences (0.607 instead of 0.637 obtained when assuming random distribution of markers).

### DNA motifs

We used MEME (version 4.6.1) [147], a software for discovering motifs in sets of DNA sequences, to search for motifs enriched in sequences around CO and GC events. We focused on 1,909 CO and 3,701 GC events delimited by 500 bp or less (CO_500_ and GC_500_). When the length of the sequence delimited by adjacent markers was less than 500 bp we extended the sequence both at the 5′ and 3′ ends up to a total of 500 bp. We searched for motifs with nucleotide size ranging between 5 and 20 nucleotides and used as a background sequence model one that takes into account both nucleotide and dinucleotide composition (first-order Markov model), the latter important to capture the observed frequency of dinucleotide repeats across the genome. MEME was applied to search for motifs on both the given DNA strand and the reverse complement strand and allowing any number of repetitions per sequence using the complete 500 bp sequences. We also used MEME to generate sequence LOGOS for each discovered motif.

Figure 6 and Figure 7 show the 18 and 10 motifs enriched in CO_500_ and GC_500_, respectively, with corrected probabilities (E-values) smaller than 10^−10^. E-values represent the expected number of sequences in a random database of the same size that would match the motifs as well as the sequence does. To obtain comparable E-values for CO and GC events we applied MEME to a random set of 1,000 CO_500_ and 1,000 GC_500_ sequences. We also investigated the commonness of the motifs with E-value<10^−10^ within CO_500_ and GC_500_ sequences (Figure S4).

### Centromeric and telomeric regions with reduced CO rates

For the sake of analyzing the distribution of recombination rates along chromosomes and their possible causes or consequences we designated centromeric and telomeric regions with visibly reduced CO (see Figure 1) as possibly influencing some of our analyses (eg, heterogeneity along chromosomes, CO *vs* GC rates, local distribution of CO and GC events, etc.). Thus most analyses were performed using whole chromosomes as well as after removing the fourth chromosome (which has no detectable CO) and these long, broadly defined peripheral regions (see Table 3).

**Table 3:** Genomic regions investigated after removing centromeric and telomeric regions.

Chromosome arm	Genomic region*
X	2.3 — 20.8 Mb
2L	0.5 — 17.4 Mb
2R	5.2 — 20.8 Mb
3L	0.7 — 19.9 Mb
3R	9.7 — 26.9 Mb

* Genomic location based on the nucleotide position in the *D. melanogaster* reference (r.5.3) genome sequence.

### Nucleotide composition across the *D. melanogaster* genome

We investigated the possible relationship between recombination rates (*c* or γ) and nucleotide composition across the *D. melanogaster* genome to test the hypothesis of a bias in heteroduplex repair with AT/GC heterozygotes being preferentially repaired towards G and C nucleotides (aka, gene conversion bias). Our analyses of nucleotide composition in the *D. melanogaster* genome were based on the *D. melanogaster* reference sequence (release 5.3, January 2011). We annotated all gene models (not only protein coding genes), transposable elements and repetitive sequences onto the reference sequence and marked any overlapping annotation.

To test for gene conversion bias in *D. melanogaster*, we focused on G+C nucleotide composition at intergenic and intronic sites using adjacent 100-kb regions. Intergenic sites were defined as those sites between any annotated gene model (thus also excluding annotated UTRs), transposable elements or repetitive sequences. Intronic sites were defined as those that are only associated with an intron definition in protein coding genes (i.e., sites that are either ‘intron’ or ‘exon’ depending on alternatively spliced forms were not considered) and do not overlap with any other annotation.

### Nucleotide polymorphism across the *D. melanogaster* genome

For the sake of obtaining information on polymorphism levels across the genome we used DGRP sequenced strains [142], all from the same natural population (Raleigh, NC, USA) than the RAL strains used in this study, and added our sequencing data from the parental RAL strains. We also included the sequenced strains MDG and PNG in the overall analysis of polymorphism. Among all DGRP strains, we chose the 34 strains with 454-Roche as well as Illumina sequencing reads to maximize SNP accuracy. DGRP sequencing reads were obtained from NCBI short read archive (SRA) study SRP000694. For each strain, we obtained reference sequences using the same mapping procedure described above now using BWA-SW [148] to map additional 454 reads. We called SNPs relative to the *D. melanogaster* genome reference when SNP quality was greater than Q40 and generated consensus sequences without ‘marking’ heterozygous sites.

We estimated nucleotide polymorphism as the pairwise nucleotide variation per site (π), with X-linked values adjusted to generate comparable estimates to autosomal regions. To test the hypothesis of a heterozygosity-dependent GC∶CO repair bias imposed after DSB formation in *D. melanogaster*, we investigated the relationship between polymorphism and the ratio γ/*c* or γ across the genome using total π. To study the relationship between polymorphism and *c* we focused on polymorphism at noncoding sites (intergenic and introns). Analyses comparing estimates of π with either γ/*c*, γ or *c* across the genome were based on values from 100-kb adjacent windows.

### Data availability

Estimates of recombination reported in this study are publicly available, for any genomic region or gene in *D. melanogaster*, from www.recombination.biology.uiowa.edu and www.recombinome.com.

## Supporting Information

Figure S1 Double-strand repair (DSB) pathway and recombination during meiosis. The DSB repair via double Holliday junction can generate either crossover (CO) or non-crossover (gene conversion, GC) events while the Holliday junction-independent repair (synthesis-dependent strand annealing, SDSA) mechanism causes only GC events.
(TIF)

Figure S2Crossing over rates (*c*) along chromosome arms based on n^th^ polynomial equations relating the physical position to *c*. A single polynomial has been applied per chromosome arm, with the physical position (*x*) in Mb and *c* (*y*) in cM/Mb. The equations best fitting adjacent 100-kb windows are the following: 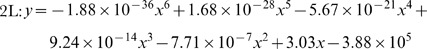


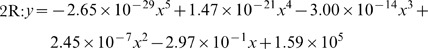


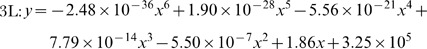


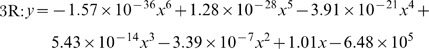


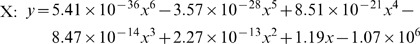

(TIF)

Figure S3 Relationship between sample mean and sample median for rates of crossing over (*c*). (A) Sample mean and sample median for *c* across the *D. melanogaster* genome estimated for adjacent 250-kb windows and shown separately for each chromosome arm. *c* indicates cM/Mb per female meiosis. (B) Blowup of the relationship between *c* based on sample mean and *c* based on sample median when *c* (sample mean) is smaller than 3 cM/Mb.
(TIF)

Figure S4 Cumulative percentage of sequences with CO and GC motifs. Percentage of sequences with one or more of the 18 CO and 10 GC motifs found to be overrepresented in CO_500_ and GC_500_ sequences, respectively (see Figure 7 and Figure 8 for details).
(TIF)

Figure S5 Schematic representation of the crossing methodology used in this study to generate recombinant chromosomes between *D. melanogaster* parental strains.
(TIF)

Table S1 24 custom-designed 7-nucleotides tags.
(DOC)
