# Supplementary figures and images for: The Many Landscapes of Recombination in Drosophila melanogaster
Source: PLoS Genet. 2012 Oct 11;8(10):e1002905. doi: 10.1371/journal.pgen.1002905 (PMC3469467; doi:10.1371/journal.pgen.1002905)

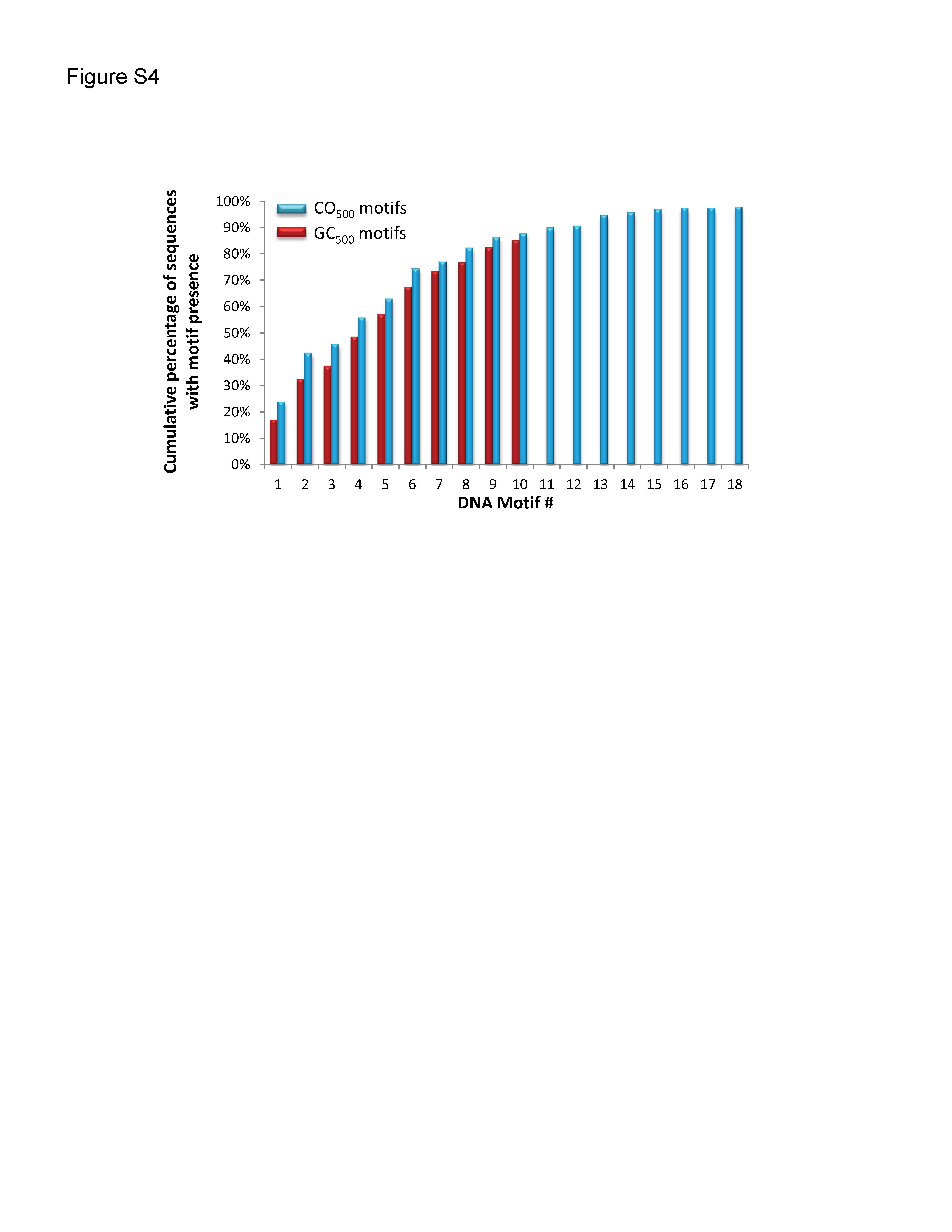

Supplement: Figure S4 — Cumulative percentage of sequences with CO and GC motifs. Percentage of sequences with one or more of the 18 CO and 10 GC motifs found to be overrepresented in CO500 and GC500 sequences, respectively (see Figure 7 and Figure 8 for details). (TIF) [file pgen.1002905.s004.tif]
