# Supplementary material for: The Many Landscapes of Recombination in Drosophila melanogaster
Source: PLoS Genet. 2012 Oct 11;8(10):e1002905. doi: 10.1371/journal.pgen.1002905 (PMC3469467; doi:10.1371/journal.pgen.1002905)
Supplement: Table S1 — 24 custom-designed 7-nucleotides tags. (DOC) [file pgen.1002905.s006.doc]

**Table S1. Custom-designed 7-nucleotides tags.**

| Tag # | Sequence |
| --- | --- |
| Tag-1 | AGATGGT |
| Tag-2 | CACGTCG |
| Tag-3 | CGAGAAG |
| Tag-4 | GATCTTG |
| Tag-5 | GTTAATC |
| Tag-6 | TCAGGAC |
| Tag-7 | TGCTAAT |
| Tag-8 | ACAGTTG |
| Tag-9 | ACTTCAG |
| Tag-10 | ATAGACA |
| Tag-11 | CAATCAA |
| Tag-12 | CTTCAGG |
| Tag-13 | GGAATGG |
| Tag-14 | GGCTTCA |
| Tag-15 | GGTGAGA |
| Tag-16 | TACCGCC |
| Tag-17 | TCTATAA |
| Tag-18 | TTGTAGA |
| Tag-19 | ACGGAGC |
| Tag-20 | ATCGTAC |
| Tag-21 | CATCGAT |
| Tag-22 | GCGCGGT |
| Tag-23 | TGTTCTC |
| Tag-24 | TCATCCT |
